# “Sex‐Based Inequities in Post‐STEMI Secondary Prevention: A Critical Appraisal and Path Forward”

**DOI:** 10.1002/clc.70252

**Published:** 2025-12-29

**Authors:** Ibadullah Tahir, Hunain Shahbaz

**Affiliations:** ^1^ Ayub Medical College Abbottabad Pakistan

Dear Editor,

Evelo et al.'s recent work examines the differences between genders in how often combination evidence‐based medicines (cEBMs) are prescribed after ST‐elevation myocardial infarction (STEMI). The authors indicate that when controlling for other variables that may account for this trend, the gap between men and women lessens. However, it seems to merit further examination since the reduced unadjusted disparity (66% vs. 72.8%) continues to be a clinical gap similar to that reported in the treatment of women who have experienced an acute coronary syndrome [1, 2, 3].

A number of factors that are unique to women (e.g., age, comorbidities) may cloud true disparities, since eliminating those factors will allow you to understand the true difference between men and women when addressing providers' failure to deliver proper care to either gender based on factors such as biological predisposition.

In addition, the single‐center design of this study reduces the extent to which the findings can be applied to the population as a whole, since it has been demonstrated multiple times through larger multi‐center studies that women are less likely than men to receive guideline‐recommended treatments and invasive interventions at a variety of healthcare facilities [1, 3, 4].

The increased prescribing rates observed in this study may be attributed to the practices of the institution rather than a trend in the overall population. Additionally, the day after discharge (DAD) measure of cEBM did not capture any changes in medications, de‐prescribing, or patient‐directed discontinuation that occurred prior to discharge. Real‐world adherence after an MI will have a significant impact on post‐MI outcomes and limited interpretation of long‐term implications. Further investigation is necessary. Women received percutaneous coronary interventions (PCI) and CABG less often compared to men, even though cardiology guidelines state that both processes are equally beneficial and that secondary preventive therapies for both genders are beneficial [5].

In Evelo et al., PCI was an independent predictor of an increased likelihood of receiving cEBM, suggesting that procedural differences may indirectly perpetuate differences in drug therapies, as well. The findings of this study, indicating a greater 6‐month rate of both stroke and death for females, are consistent with the male‐to‐female ratio of outcomes reported in prior studies [1, 6, 7]. Although it is not possible to draw causal conclusions from this data, there is a need to investigate whether any of the clinically modifiable parameters, including intensity of treatment, clinician bias, or avoidance of prescribing due to perceived risk, contribute to the downstream sex disparity in outcomes.

Evelo et al. make an important first step in this area; however, there are many ways to build upon this foundation through continued research. Future studies should also consider mixed‐methods approaches, incorporating physician interviews as well as the patients' experience with cEBM to understand the impact of communication, shared decision‐making, and diagnostic uncertainty on the use of cEBM. Additionally, the use of implementation science tools, such as default prescribing bundles, audit‐feedback cycles, or automated discharge reminders, may provide potential solutions to the system‐level barriers that impact cEBM. Finally, including multicenter and/or international cohorts in future studies will allow researchers to evaluate the robustness of the finding of no differences in sex across diverse clinical environments. Evaluating the differences in drug tolerability, when men versus women stop taking medication early, and long‐term medication adherence can help us understand the factors contributing to these inequities.

In support of this conclusion, while Evelo et al.'s article provides us with valuable insight regarding the prescribing patterns of cEBMs in STEMI patients, it is important that we take a closer look at other aspects (structural, behavioral, and system‐related) to identify and address any underlying contributors to these inequities. Therefore, future research should incorporate an examination of patient, provider, and health system factors to provide equitable access to secondary prevention methods that conform with national guidelines for all survivors of STEMI.

## Author Contributions

All authors have read and approved the final version of the manuscript.

## Funding

The authors received no specific funding for this work.

## Ethics Statement

The authors have nothing to report.

## Consent

The authors have nothing to report.

## Conflicts of Interest

The authors declare no conflicts of interest.

## Data Availability

The authors have nothing to report.
